# Impaired kidney function is associated with lower cognitive function in the elder general population. Results from the Good Aging in Skåne (GÅS) cohort study

**DOI:** 10.1186/s12877-019-1381-y

**Published:** 2019-12-19

**Authors:** Tomas Månsson, Marieclaire Overton, Mats Pihlsgård, Sölve Elmståhl

**Affiliations:** Department of Clinical Sciences in Malmö, Division of Geriatric Medicine, Lund University and Skåne University Hospital, Jan Waldenströms gata 35, 205 02 Malmö, Sweden

**Keywords:** Kidney, Renal, Function, GFR, “Glomerular filtration”, Impairment, Decline, Cognitive, Cognition, Domains

## Abstract

**Background:**

A possible connection on vascular basis between impaired kidney function and cognitive dysfunction has been suggested in previous studies. Contradictory results regarding specific cognitive domains have been reported. The aim for this study was to investigate the association between kidney function and specific cognitive domains.

**Methods:**

In this cross-sectional design, data from the general population based cohort study “Good aging in Skåne” (GÅS) was used. The sample included 2931 subjects ages 60 to 93 randomly selected from the southern part of Sweden. Estimated glomerular filtration rate (eGFR) for both creatinine and cystatine C was calculated using the chronic kidney disease epidemiology collaboration (CKD-EPI) equation. The subjects underwent a test battery of neuropsychological tests assessing global cognitive function, learning and memory, language, complex attention, executive function, perceptual motor and meta-memory. Adjustments were made for age, sex, education and country of origin.

**Results:**

After adjustment for demographic variables, impaired kidney function was associated with 0.41 points worse result in MMSE, 0.56 points worse result in recognition, 0.66 points worse result in word fluency, 0.45 points worse result in digit cancellation, 0.99 points worse result in pattern comparison, and 3.71 s longer time to finish TMT B-A. Associations to cognitive function was also noted for mildly impaired kidney function defined as eGFR 45- < 60 ml/min/1,73m^2^. No association was found between kidney function and meta-memory.

**Conclusions:**

Impaired kidney function as well as the severity of impaired kidney function is associated with impairment in learning and memory, language, complex attention, executive function and global cognitive function, but not meta-memory.

## Background

Impaired kidney function and cognitive dysfunction are very common conditions in the elderly population [1–3]. The prevalence of impaired kidney function – defined as glomerular filtration rate (GFR) < 60 ml/min/1,73m^2^ [4, 5] – has been estimated to 23–36% in individuals ≥65 years of age [1], and increases immensely by age to about 55 to 75% in individuals > 80 years of age [6]. In Europe, the prevalence of mild cognitive impairment (MCI) has been estimated to > 20% in individuals ≥85 years of age, and the prevalence of dementia has been estimated to increase from 1% in the age group 65–70 years to 45% in individuals ≥95 years of age [3].

Since previous studies indicate a possible connection between impaired kidney function and cognitive dysfunction [7, 8], impaired kidney function is a known risk factor for vascular disease [9], and cerebrovascular disease is common when cognitive dysfunction is present [10], a potential connection on vascular basis between impaired kidney function and cognitive dysfunction has been suggested [11]. Previous studies investigating the connection between impaired kidney function and declined function in different cognitive domains have shown decline in multiple and various cognitive domains [12], although executive function stands out in some studies [11, 13]. Impairment in multiple cognitive domains have been linked to cerebrovascular disease, although executive function and the speed of processing tend to be more associated [14, 15]. This study aims to investigate if there is an association between GFR and cognitive function in the general elder population, and if so, which specific cognitive domains are associated.

## Methods

This is a cross-sectional study of data from the general population based cohort study” Good Aging in Skåne” (GÅS), being performed at the Department of Geriatric Medicine, Skåne University Hospital, Sweden [16]. In total 2931 subjects 60 to 93 years of age living in five municipalities in urban and rural areas in the south of Sweden were included from February 2001 to July 2004. They were randomly selected from the national Swedish population register. Home visits were offered to subjects unable to visit the research center. The participants underwent a comprehensive health examination by a physician, registered nurse and psychological test administrator, including medical examination, medical history, physical examination, neuropsychological testing, anthropometrics, interview and self-reported questionnaires and biobanking. The participants’ mean age at baseline was 71.5 years (SD 10.3 years). The participation rate at baseline was 60%. Information on diseases was based on medical records, medical history and examination by a physician based on the ICD-10 and DSM-IV criteria for dementia.

### Kidney function

Blood samples were taken nonfasted by a nurse and cryopreserved at baseline. Cystatin C was analyzed as one batch in 2007 by hospital laboratory using Gentians reagent with a Beckman Coulter LX 20. Creatinine was also analyzed as one batch the same year by hospital laboratory using a modified Jaffe method with a Beckman Coulter LX 20 traceable to isotope-dilution mass spectrometry (IDMS) [17]. Estimated glomerular filtration rate (eGFR) for both creatinine and cystatine C was calculated using the well-established and reliable chronic kidney disease epidemiology collaboration (CKD-EPI) equation [18]. The mean for eGFR_crea_ and eGFR_cyst_ was used, since this mean has been proven more reliable than either estimate separate [19].

### Cognitive function

The participants underwent a test battery of neuropsychological tests. The tests were performed during 1.5 h conducted by a study trained test administrator with a bachelor’s degree in behavioral sciences. The tests included the cognitive domains complex attention, executive function, learning and memory, language, perceptual-motor, described in the widely accepted and frequently applied DSM-5 [20]. Beyond these cognitive domains, global cognitive function and meta-memory were also tested.

### *Global cognitive function* was assessed using mini mental state examination (MMSE) [21]

*Immediate memory*, a subdomain to the cognitive domain *learning and memory* in DSM-5 [20], was assessed using the digit span forward test [22]. The participant was asked to repeat a number combination between 2 and 8 numbers ranging from 1 to 9. The longest correct recalled digit span was used for assessment.

*Recent memory*, a subdomain to the cognitive domain *learning and memory* in DSM-5 [20], was assessed using the tests free recall and recognition [23]. In the test free recall 16 unrelated words were presented to the participant. The participant then had 2 min to freely recall as many words he/she could remember. The number of correct recalled words was used for assessment. In the recognition test the 16 words from the test free recall were presented again mixed with 16 new unrelated words. The task for the participant was to identify which words had been presented in the test free recall. The number of correct recognized words minus the number of incorrect words (false hits) was used for assessment.

*Expressive language*, a subdomain to the cognitive domain *language* in DSM-5 [20], was assessed using the tests word fluency F and A and word fluency animals and occupations [22]. In word fluency F and A, the participant was assigned to name as many words he/she could come up with that started with the letter F and then the letter A. 1 min was given for each letter. For word fluency animals and occupations, the participants were instructed to name as many animals as possible in 1 min and then as many occupations as possible in 1 min. The mean for the number of words from F and A, and animals and occupations, was used for assessment.

*The speed of processing*, a subdomain to the cognitive domain *complex attention* in DSM-5 [20], was assessed using the tests digit cancellation [24] and pattern comparison [25]. In digit cancellation, 11 rows of random numbers between 1 and 9 was presented to the participant on a piece of paper. The task was to draw a line over as many fours as possible during 30 s. The number of correct lines was used for assessment. For pattern comparison columns with figures in pairs were presented to the participant on a piece of paper. The task was, during 2 × 30 s, to decide if the figures in each pair was identical or not. The number of correct answers was used for assessment.

*Mental flexibility*, a subdomain to the cognitive domain *executive function* in DSM-5 [20], was assessed using the trail making test (TMT) A and B [22]. In TMT A the participant was instructed to draw lines in numeric order between circles containing numbers on a piece of paper (1–2-3 and so on). In TMT B the participant was instructed to draw lines in the same way, but in this test the circles contained both numbers and letters. The task was to draw lines between the circles containing numbers and letters alternating between numeric and alphabetic order (1-A-2-B and so on). The participants had no time limit. Participants who had one error or more in either TMT A or TMT B were excluded. When individuals finished TMT A faster than 7 s or TMT B faster than 12 s, that is > two SD from mean, suspicion of misprint in documentation was evoked, and those individuals were excluded. In total 595 individuals were excluded from the test. In order to measure mental flexibility, but avoid measuring the speed of perception, the time it took for the participant to finish TMT A was subtracted from the time it took to finish TMT B (TMT B-A) and used for assessment [26].

*Working memory*, a subdomain to *executive function* in DSM-5 [20], was assessed using the digit span backwards test [27]. In this oral test the participant was instructed to repeat a number combination of 2–8 numbers ranging from 1 to 9 backwards. The best result was used for assessment.

*Visual perception*, a subdomain to the *perceptual-motor* domain in DSM-5 [20], was assessed using the mental rotations test. The test was a simplified version, with 10 assignments [28], of the Shepard-Metzler test [29]. In each assignment a 3-dimensional figure of cubes was presented to the left on a piece of paper, with 3 rotated figures to the right. Only one of the 3 rotated figures was identical to the figure to the left. The assignment was to choose the identical, but rotated figure. The participant was given 45 s for each assignment. The number of correct answers divided with the number of answered assignments was used for assessment.

*Meta-memory* was assessed using a confidence judgement test [30]. In this test the participant was presented with 10 general questions on a piece of paper and was assigned to choose 1 of 2 written answers to each question. The questions were of the following kind:” From which language does the word” alcohol” originate? A Greek B Arabic”. The participant thereafter was assigned to report how” certain” he/she was of have answered each of the questions correctly, by choosing a denary in percent between 50 and 100%, where 50% represented total uncertainty and 100% represented complete certainty of having answered correctly. The following calibration formula, described by Dahl et al. [24], was used to estimate the confidence:

$$ \frac{1}{n}{\sum}_{t=1}^T nt\left( rt- ct\right)2, $$

where *n* represents the total number of answered questions, *T* represents the number of confidence levels (6 in this case), *nt* represents the number of times the confidence level *rt* was reported, and *ct* represents the portion of correct answers to the total number of answers where confidence level *rt* was reported. If the calibration value from the formula is 0, the calibration is perfect, that is, the confidence of the participant of having answered correctly is in line with how correctly the participant in fact did answer. The bigger the confidence value from the formula, the more the participant is misjudging his/her ability to answer the questions correctly.

### Covariates

Adjustments were made for age, sex, education and country of origin. Education was categorized as elementary school not completed, fulfilled elementary school, fulfilled secondary school, and one year or more of higher education or university degree. Country of origin was categorized as born in Sweden and born in other country than Sweden. Description of covariates are presented in Table 1.

**Table 1:** Characteristics of the study sample

Variable			Groups divided by kidney function			*p*-value	All participants
	eGFR < 30 mL/min/1.73 m^2^	eGFR 30- < 45 mL/min/1.73 m^2^	eGFR 45- < 60 mL/min/1.73 m^2^	eGFR < 60 mL/min/1.73 m^2^	eGFR ≥60 mL/min/1.73 m^2^		
Number (%)	70 (2.9)	241 (9.9)	483 (19.9)	794 (32.7)	1637 (67.3)		2431
Age (years)	83.0 ± 8.5	83.8 ± 6.8	78.9 ± 8.3	80.9 ± 8.2	66.9 ± 7.8	< 0.001	71.4 ± 10.3
Sex no. (%)						< 0.001	
women	37 (52.9)	156 (64.7)	294 (60.9)	487 (61.3)	837 (51.1)		1324 (54.5)
men	33 (47.1)	85 (35.3)	189 (39.1)	307 (38.7)	800 (48.9)		1107 (45.5)
Education no. (%)						< 0.001	
elementary school not completed	1 (1.4)	8 (3.3)	20 (4.1)	29 (3.7)	41 (2.5)		70 (2.9)
elementary school	41 (58.6)	147 (61.0)	277 (57.3)	465 (58.6)	760 (46.4)		1225 (50.4)
secondary school	17 (24.3)	67 (27.8)	128 (26.5)	212 (26.7)	470 (28.7)		682 (28.1)
> = 1 year extra or university degree	11 (15.7)	19 (7.9)	58 (12.0)	88 (11.1)	366 (22.4)		454 (18.7)
Country of origin no. (%)						< 0.001	
Sweden	65 (92.9)	232 (96.3)	445 (92.1)	742 (93.5)	1441 (88.0)		2183 (89.8)
other than Sweden	5 (7.1)	9 (3.7)	38 (7.9)	52 (6.5)	196 (12.0)		248 (10.2)
Living no. (%)						0.810	
in urban environment	58 (82.9)	210 (87.1)	442 (91.5)	710 (89.4)	1469 (89.7)		2179 (89.6)
in rural environment	12 (17.1)	31 (12.9)	41 (8.5)	84 (10.6)	168 (10.3)		252 (10.4)
Cardiovascular risk factors							
Hypertension no. (%)						< 0.001	
Yes	37 (53.6)	112 (46.9)	189 (39.3)	338 (42.8)	330 (20.2)		668 (27.6)
No	32 (46.4)	127 (53.1)	292 (60.7)	451 (57.2)	1303 (79.8)		1754 (72.4)
Smoking no. (%)						< 0.001	
Active smoker	7 (10)	13 (5.4)	61 (12.7)	81 (10.2)	341 (20.9)		422 (17.4)
Former smoker	35 (50)	87 (36.1)	168 (34.9)	290 (36.6)	642 (39.3)		932 (38.4)
Never smoked	28 (40)	141 (58.5)	253 (52.5)	422 (53.2)	651 (39.8)		1073 (44.2)
Diabetes type 1 no. (%)						0.813	
Yes	2 (2.9)	4 (1.7)	1 (0.2)	7 (0.9)	13 (0.8)		20 (0.8)
No	67 (97.1)	236 (98.3)	478 (99.8)	781 (99.1)	1621 (99.2)		2402 (99.2)
Diabetes type 2 no. (%)						0.011	
Yes	8 (11.4)	20 (8.3)	38 (7.9)	66 (8.3)	92 (5.6)		158 (6.5)
No	62 (88.6)	220 (91.7)	445 (92.1)	727 (91.7)	1544 (94.4)		2271 (93.5)

Characteristics of the Good Aging in Skåne study sample in relation to kidney function; severely impaired (eGFR < 30 mL/min/1.73 m^2^), moderately impaired (eGFR 30- < 45 mL/min/1.73 m^2^), mildly impaired (eGFR 45- < 60 mL/min/1.73 m^2^) and normal kidney function (eGFR ≥60 mL/min/1.73 m^2^)

Distribution of the variables was tested between the groups with eGFR ≥60 and < 60 mL/min/1.73 m^2^. Pearson Chi-Square test was used for all variables except for age, where Mann-Whitney test was used

Values above represent number of participants. Values within parenteses represent percentage. Values following ± represent SD

Abbreviations: *eGFR* estimated glomerular filtration rate, *SD* Standard deviation

### Exclusion

Depression is associated with cognitive impairment [31]. The comprehensive psychopathological rating scale (CPRS) was used to detect depression. A CPRS score above 20 indicated depression [32, 33]. Fourty-two participants met this criterion. Two hundred fourty individuals had not answered > 2 questions in the CPRS-questionnaire. Twenty-seven individuals (9,2 ^0^/_00_) had left only1–2 questions unanswered in the CPRS-questionnaire. These unanswered questions were imputed with the mean value of the answered CPRS-questions for each of these 27 participants individually.

A physician identified individuals with a prior diagnosis of dementia or who met the criteria for dementia defined in DSM-IV [34]. The information was based on medical records, clinical examination, and proxy information from family members/relatives and ward staff. Two hundred individuals met the criteria for dementia or had insufficient data for an assessment to be made.

One hundred sixteen individuals had missing blood samples. One hundred seventy-four individuals did not participate in the cognitive tests. One hundred nineteen individuals had missing information on education.

**Fig. 1:**
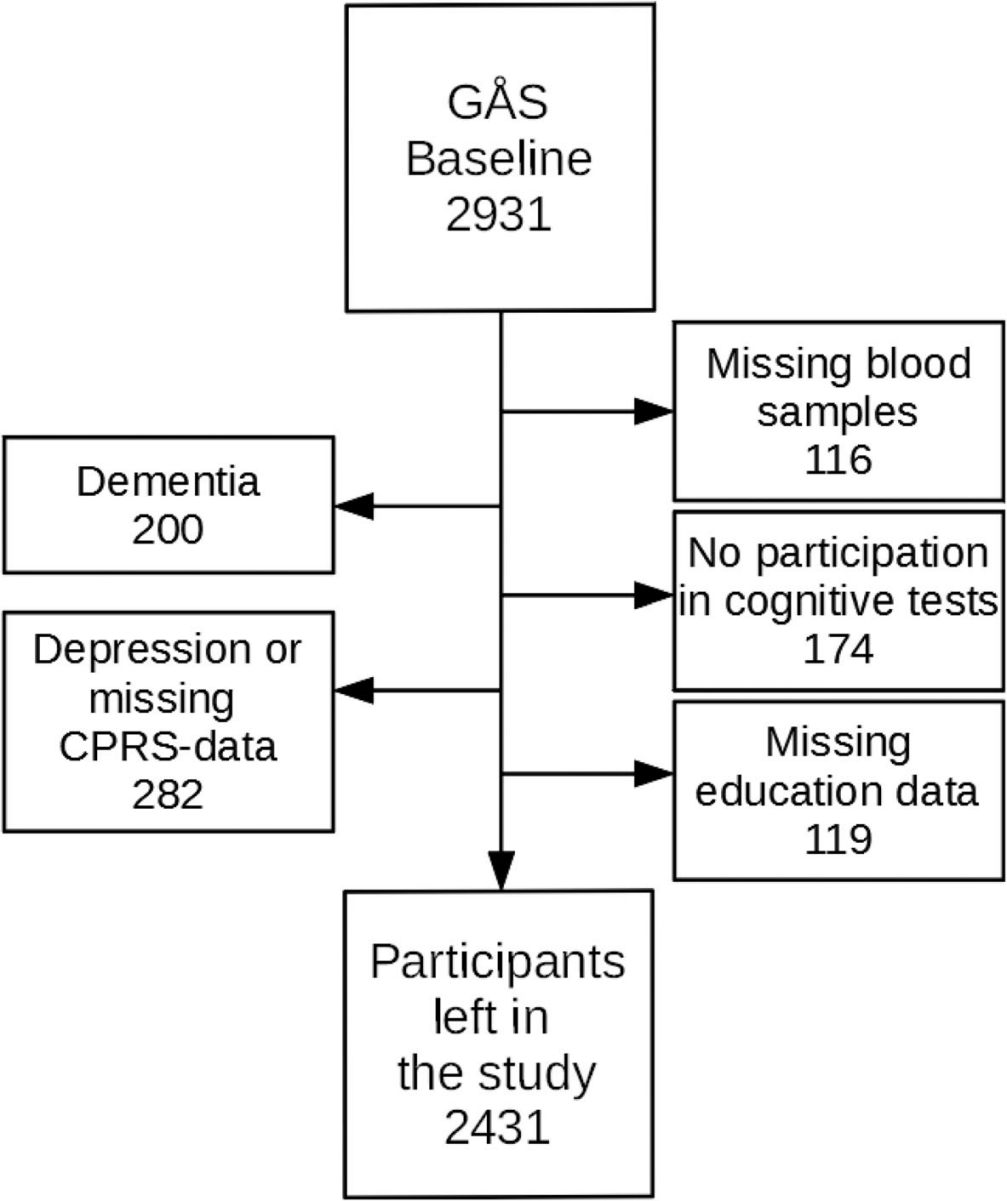
Number of participants

In total, 500 individuals were excluded due to depression, and/or dementia, and/or missing information on cognitive tests, kidney function and/or education, leaving 2431 individuals remaining in the study sample, see Fig. 1. The excluded individuals tended to be older, more often of female sex, had less education and were more often born in Sweden than another country, see Additional file 1.

A flow chart of study sample from the Good Aging in Skåne (GÅS) general cohort study. The numbers are given for participants at baseline in GÅS, the number of participants meeting the different exclusion criteria, and the number of participants left in the present study. Several participants met multiple exclusion criteria.

### Statistics

The data for all cognitive tests, except MMSE, TMT B-A and confidence judgement, were normally distributed, with skewness and kurtosis +/− 2. The group sizes for MMSE, TMT B-A and confidence judgement were big enough though, to allow use of independent samples t-tests [35]. Independent samples t-tests were used for all cognitive tests to compare the results for individuals with impaired kidney function (eGFR < 60 mL/min/1.73 m^2^) to individuals with normal kidney function (eGFR ≥60 mL/min/1.73 m^2^).

Multiple linear regression models were used to compare the results of the cognitive tests for participants based on their kidney function categorized in impaired and normal function, and also in relation to severity of impaired kidney function divided into four groups (eGFR levels/min/1.73 m^2^; < 30 mL, 30- < 45 mL, 45- < 60 mL and ≥ 60 mL). All analyses were adjusted for age, sex, education and country of origin. The same analyses were performed after stratification for age into four age groups (60–69 years, 70–79 years, 80–89 years and > 90 years).

Multiple linear regression models including interaction analyses were used to detect interaction between kidney function and the demographic variables above, see Additional files 4 and 5.

## Results

Individuals with impaired kidney function (eGFR < 60 mL/min/1.73 m^2^) had lower cognitive test results in all the cognitive tests except confidence judgement, compared to individuals with normal kidney function (eGFR ≥60 mL/min/1.73 m^2^), see Table 2. After adjustments for age, sex, education and country of origin, lower cognitive results remained for MMSE, recognition, word fluency, digit cancellation, pattern comparison and TMT B-A, as presented in Table 3.

**Table 2:** Results of the cognitive tests in relation to two groups based on eGFR

Cognitive test	Cognitive domain	Number of0020participants by eGFR		Test result by eGFR		Mean difference	95% CI of difference		*P*-value
									(2-tailed)
		eGFR < 60 mL/min/1.73 m^2^	eGFR ≥60 mL/min/1.73 m^2^	eGFR < 60 mL/min/1.73 m^2^	eGFR ≥60 mL/min/1.73 m^2^		Lower	Upper	
MMSE	Global	778	1624	25.84 ± 3.08	27.34 ± 2.32	−1.5	− 1.74	−1.25	< 0.001
Digit span forward	Learning and memory	763	1616	5.95 ± 1.74	6.44 ± 1.86	−0.48	− 0.63	− 0.33	< 0.001
Free recall	Learning and memory	734	1595	5.77 ± 2.30	7.05 ± 2.35	− 1.28	− 1.48	− 1.07	< 0.001
Recognition	Learning and memory	711	1588	10.85 ± 3.49	11.88 ± 2.81	−1.03	− 1.32	− 0.74	< 0.001
Word fluency	Language	765	1611	10.73 ± 4.58	12.51 ± 4.93	−1.77	−2.18	− 1.37	< 0.001
Digit cancellation	Complex attention	711	1608	14.56 ± 4.17	17.75 ± 4.01	−3.18	−3.54	−2.83	< 0.001
Pattern comparison	Complex attention	692	1598	21.33 ± 7.20	28.22 ± 7.36	−6.9	−7.55	−6.25	< 0.001
TMT B-A	Executive function	499	1337	26.00 ± 23.38	15.12 ± 14.41	10.88	8.69	13.08	< 0.001
Digit span backwards	Executive function	760	1613	4.87 ± 1.84	5.54 ± 1.97	− 0.67	− 0.83	− 0.5	< 0.001
Mental rotations	Perceptual-motor	682	1580	0.56 ± 0.17	0.62 ± 0.19	− 0.06	− 0.07	− 0.04	< 0.001
Confidence judgement	Meta-memory	749	1594	0.11 ± 0.08	0.11 ± 0.08	− 0,005	− 0.01	0,002	0.185

Results of the cognitive tests in relation to impaired kidney function (eGFR < 60 mL/min/1.73 m^2^) and normal kidney function (eGFR ≥60 mL/min/1.73 m^2^)

Some participants did not participate in all of the cognitive tests. Every participant performed at least one of the cognitive tests

Equal variances assumed for free recall, digit cancellation, pattern comparison and confidence judgement

Equal variances not assumed for MMSE, digit span forward, recognition, word fluency, TMT B-A, digit span backwards and mental rotations

Values following ± represent SD

Abbreviations: *eGFR* estimated glomerular filtration rate, *CI* Confidence interval, *SD* Standard deviation

**Table 3:** Results of the cognitive tests in relation to two and four groups based on eGFR

(A) Kidney function by two groups						
Cognitive test	Cognitive domain	Number of participants	Mean test results	B-coefficient	95% CI for B	*p*-value
MMSE	Global	2402	26.86	− 0.411	− 0.682, 0.140	0.003
Digit span forward	Learning and memory	2379	6.28	−0.037	− 0.232, 0.158	0.708
Free recall	Learning and memory	2329	6.64	−0.239	−0.482, 0.005	0.054
Recognition	Learning and memory	2299	11.56	−0.562	−0.900, − 0.224	0.001
Word fluency	Language	2376	11.94	−0.663	−1.148, − 0.179	0.007
Digit cancellation	Complex attention	2319	16.77	−0.45	−0.869, − 0.031	0.035
Pattern comparison	Complex attention	2290	26.14	−0.988	−1.697, − 0.279	0.006
TMT B-A	Executive function	1836	18.08	3.707	1.539, 5.874	0.001
Digit span backwards	Executive function	2373	5.33	−0.159	−0.365, 0.047	0.131
Mental rotations	Perceptual-motor	2262	0.6	−0.001	−0.021, 0.019	0.91
Confidence judgement	Meta-memory	2343	0.11	0.003	−0.006, 0.012	0.546
(B) Kidney function by four groups						
Cognitive test	Cognitive domain	Number of participants	eGFR level compared to eGFR ≥60 mL/min/1.73 m^2^ as a reference	B-coefficient	95% CI for B	*p*-value
MMSE	Global	2402	< 30 30- < 45 45- < 60	−0.788 −0.776 −0.246	−1.412, − 0.164 −1.173, − 0.379 − 0.538, 0.046	0.013 < 0.001 0.099
Digit span forward	Learning and memory	2379	< 30 30- < 45 45- < 60	− 0.119 − 0.112 − 0.003	− 0.569, 0.331 − 0.400, 0.175 − 0.214, 0.207	0.603 0.444 0.976
Free recall	Learning and memory	2329	< 30 30- < 45 45- < 60	−0.341 − 0.436 − 0.162	−0.925, 0.243 − 0.798, − 0.075 − 0.425, 0.101	0.252 0.018 0.226
Recognition	Learning and memory	2299	< 30 30- < 45 45- < 60	− 0.985 − 0.809 − 0.433	− 1.790, − 0.179 − 1.312, − 0.306 − 0.799, − 0.066	0.017 0.002 0.021
Word fluency	Language	2376	< 30 30- < 45 45- < 60	−0.533 − 0.832 − 0.620	−1.675, 0.610 − 1.542, − 0.121 − 1.143, − 0.097	0.361 0.022 0.020
Digit cancellation	Complex attention	2319	< 30 30- < 45 45- < 60	−0.307 − 0.744 − 0.371	− 1.321, 0.706 − 1.375, − 0.112 − 0.823, 0.081	0.553 0.021 0.108
Pattern comparison	Complex attention	2290	< 30 30- < 45 45- < 60	−0.577 −2.121 − 0.672	−2.303, 1.148 −3.194, − 1.048 − 1.435, 0.091	0.512 < 0.001 0.084
TMT B-A	Executive function	1836	< 30 30- < 45 45- < 60	2.479 4.993 3.423	−3.202, 8.161 1.612, 8.373 1.081, 5.765	0.392 0.004 0.004
Digit span backwards	Executive function	2373	< 30 30- < 45 45- < 60	−0.303 − 0.309 − 0.094	−0.777, 0.172 − 0.614, − 0.004 − 0.317, 0.128	0.211 0.047 0.404
Mental rotations	Perceptual-motor	2262	< 30 30- < 45 45- < 60	0.016 − 0.003 − 0.002	−0.033, 0.065 − 0.033, 0.027 − 0.024, 0.019	0.530 0.840 0.845
Confidence judgement	Meta-memory	2343	< 30 30- < 45 45- < 60	0.003 0.004 0.002	−0.019, 0.024 − 0.010, 0.017 − 0.007, 0.012	0.796 0.572 0.628

Multiple linear regression models of cognitive tests in relation to kidney function, (A) divided into two groups, impaired kidney function (eGFR < 60 mL/min/1.73 m^2^) and normal kidney function (eGFR ≥60 mL/min/1.73 m^2^), with eGFR ≥60 mL/min/1.73 m2 as reference, (B) divided into four groups, severely impaired (eGFR < 30 mL/min/1.73 m^2^), moderately impaired (eGFR 30- < 45 mL/min/1.73 m^2^), mildly impaired (eGFR 45- < 60 mL/min/1.73 m^2^), and normal kidney function (eGFR ≥60 mL/min/1.73 m^2^), with eGFR ≥60 mL/min/1.73 m^2^ as reference

All analyses were adjusted for age, sex, education and country of origin

Abbreviations: *eGFR* estimated glomerular filtration rate, *CI* Confidence interval

The participants were divided into four groups depending on the severity of kidney function (eGFR levels/min/1.73 m^2^; < 30 mL, 30- < 45 mL, 45- < 60 mL and ≥ 60 mL, with normal kidney function, eGFR ≥60 mL/min/1.73 m^2^, as reference). Adjustments were made for age, sex, education and country of origin. eGFR < 30 mL/ min/1.73 m^2^ was associated with worse results in MMSE and recognition. eGFR 30- < 45 mL/min/1.73 m^2^ was associated with worse results in MMSE, free recall, recognition, word fluency, digit cancellation, TMT B-A and digit span backwards. eGFR 45- < 60 mL/min/1.73 m^2^ was associated with worse results in recognition, word fluency and TMT B-A, see Table 3.

The association of cognitive test results and impaired kidney function (eGFR < 60 mL/min/1.73 m^2^) was tested with stratification made for age into four age groups, with adjustments made for sex, education and country of origin. In the youngest age group, 60–69 years, impaired kidney function was associated with lower test score results for MMSE, recognition, digit cancellation, pattern comparison, TMT B-A and mental rotations. In the age group 70–79 years, an association was found for MMSE, free recall, word fluency and pattern comparison. An association between impaired kidney function and digit span forward was found in the oldest age group ≥90 years. No other association was found in the two oldest age groups, see Additional file 2.

The association of cognitive test results and the severity of impaired kidney function with eGFR divided into four groups with eGFR ≥60 mL/min/1.73 m^2^ as reference as above, was also tested with stratification made for age into four age groups. Adjustments were again made for sex, education and country of origin. For MMSE an association was found in the age group 60–69 years with eGFR 45- < 60 mL/min/1.73 m^2^, and in the age group 70–79 years with eGFR 30- < 45 mL/min/1.73 m^2^. For digit span forward an association was found in the age group 70–79 years with eGFR 45- < 60 mL/min/1.73 m^2^ and in the age group ≥90 years with eGFR 45- < 60. For free recall an association was found in the age group 70–79 years with eGFR 30- < 45 mL/min/1.73 m^2^. For recognition an association was found in the age group 60–69 years with eGFR 30- < 45 and 45- < 60 mL/min/1.73 m^2^. For word fluency an association was found in the age group 60–69 with eGFR 30- < 45 mL/min/1.73 m^2^. For digit cancellation an association was found in the age group 60–69 years with eGFR 30- < 45 and 45- < 60 mL/min/1.73 m^2^. For pattern comparison an association was found in the age group 60–69 years with eGFR 30- < 45 and 45- < 60 mL/min/1.73 m^2^, in the age group 70–79 years with eGFR 30- < 45 mL/min/1.73 m^2^ and in the age group 80–89 years with eGFR 30- < 45 mL/min/1.73 m^2^. For TMT B-A an association was found in the age group 60–69 years with eGFR 45- < 60 mL/min/1.73 m^2^ and in the age group 70–79 years with eGFR 30- < 45 mL/min/1.73 m^2^. For mental rotations an association was found in the age group 60–69 years with eGFR 45- < 60. No association was found for digit span backwards and confidence judgement, see Additional file 3.

Regarding the cognitive tests MMSE, word fluency, digit span backwards, mental rotations and confidence judgement, interactions were found between impaired/normal kidney function and various demographic variables, see Additional file 4. New analyses for these cognitive tests were performed, including the interactions found, as presented in Additional file 5. With consideration taken for interactions, an association between impaired kidney function and test result on the digit span backwards test, that had not been seen without consideration taken for interactions, was found.

## Discussion

This cross-sectional study showed an association between impaired kidney function as well as the severity of impaired kidney function and the cognitive domains learning and memory, language, complex attention, executive function, as well as global cognitive function, after adjustment for demographic covariates. This association was noted also for mildly impaired kidney function defined as eGFR 45–60 for learning and memory, language and executive function. The lack of association in the most severely impaired eGFR group, was most likely explained by low power, since only 70 individuals had eGFR < 30 mL/min/1.73 m^2^, compared to 241 with eGFR 30- < 45 mL/min/1.73 m^2^ and 483 with eGFR 45- < 60 mL/min/1.73 m^2^.

An association between kidney function and visual perception was seen only in the youngest age group. No association was found between kidney function and meta-memory.

The finding of an association between kidney function and multiple cognitive domains seen in this study is in line with previous studies on the subject, supporting a relationship between kidney function and multiple and various cognitive domains, as described by Elias et al. in a large review analyzing 5 cross-sectional and 10 longitudinal studies [12].

Previous studies show that impairment in multiple cognitive domains are associated with cerebrovascular disease, although executive function and the speed of processing could be more heavily associated than other domains [13, 14]. Our findings on a connection between kidney function and multiple cognitive domains, including executive function and the speed of processing, do not contradict, but rather support, a possible connection on vascular basis between impaired kidney function and cognitive dysfunction. A plausible assumption is that cerebral small vessel disease (CSVD) could constitute a large proportion of this assumed vascular denominator, since CSVD is heavily linked to cognitive impairment and has been estimated to cause up to 45% of dementia and about 20% of all stroke worldwide [36]. In regards to the special connection between executive function or speed of processing and kidney function, we did find an association. However, it is problematic to conclude if these associations are larger than the associations for other cognitive domains due to differences in the psychometric properties of the tests.

Adjustments were made for relevant demographic covariates potentially influencing cognition. Interaction analyses between eGFR and each of the demographic covariates were made. The interactions found are presented in Additional file 5. These interactions are generally hard to interpret, since the effect of normal kidney function seems to be positive for some level(s) of the interacting variable, but negative for other(s). For example, the effect of normal kidney function for those born in Sweden is 0,5 points on the MMSE test, while for foreign-born participants the opposite effect is observed. The same pattern is seen for word fluency, digit span backwards and mental rotations, raising the question if the interactions seen in this study are mainly due to chance (multiple testing).

An interesting finding was the lack of association between reduced kidney function and meta-memory, as described above, suggesting that the awareness of reduced function in memory is not affected in the presence of reduced kidney function. This is new knowledge since no previous study has investigated the association between reduced kidney function and meta-memory.

Another interesting finding was a stronger association found between kidney function and cognitive function in the younger age groups. This can probably at least partly be explained due to lack of power, though only 92–131 participants performed the different cognitive tests in the oldest age group (≥ 90 years), compared to 1229–1255 participants in the youngest age group (60–69 years). Lack of power can however probably not explain the difference between the second youngest age group (70–79 years) where 445–464 participants performed the cognitive tests compared to 489–552 participants in the second oldest age group (80–89 years).

The main strength of this study is the large population sample including both urban and rural areas, thereby increasing generalization of data. Although home visits were offered in order to reduce selection bias of healthier individuals, under-representation of elder and more frail subjects cannot be excluded. This might introduce an underestimation of noted associations.

Another strength was the large number of cognitive tests used. The cognitive domains were assessed with established standardized tests and standardized administration to ensure consistency in test scoring. Moreover, an additional strength was that multiple tests were used to assess the same specific cognitive domain, which showed similar results. The total variance of test scores for five of the cognitive tests determined by the test administrator was between 1.4 to 3.5% [37]. This further supports that the cognitive tests were carried out in a standardized way.

Earlier studies investigating the relationship between kidney function and executive function using TMT to assess executive function, commonly have used TMT B [38, 39]. A strength in our study, was using the difference between TMT B and A (TMT B-A), to measure mental flexibility (executive function), but in the same time avoid measuring the speed of processing [26].

A further strength in this study was estimating kidney function by use of both cystatine C and creatinine derived GFR analyses from one and the same batch providing a more reliable estimate than separate analyses of one of the two [19].

A weakness in this study was that no test assessed the cognitive domain social cognition [20].

The cardiovascular risk factors hypertension, smoking and diabetes type 2 were not proportionally distributed between the group with impaired kidney function and the group with normal kidney function, see Table 1. Adjustments for cardiovascular risk factors were not done in the analyses, adding a possible weakness to the study.

Another weakness is that the data was collected in 2001–2004. We cannot exclude the plausible association between kidney function and cognitive function since then has decreased somewhat, due to advancements in cardiovascular primary and secondary prophylaxis.

## Conclusions

Impaired kidney function as well as the severity of impaired kidney function is associated with impairment in learning and memory, language, complex attention, executive function and global cognitive function, but not meta-memory. The multiple cognitive domains affected in association with impaired kidney function might be related to a common denominator, such as cerebral small vessel disease. Further longitudinal studies and MRI studies are needed to explore possible predictors and causality for cognitive decline related to kidney function.

## Supplementary information

**Additional file 1.** Characteristics of included individuals versus drop outs in the study.

**Additional file 2.** Results of the cognitive tests in relation to two groups based on eGFR and age.

**Additional file 3.** Results of the cognitive tests in relation to four groups based on eGFR and age.

**Additional file 4.** Interaction analysis model.

**Additional file 5.** Results of the cognitive tests in relation to two groups based on eGFR, including interaction.

## Data Availability

The data that support the findings of this study are available from Division of Geriatric Medicine, Lund University (PI Sölve Elmståhl), but restrictions apply to the availability of these data, which were used under license for the current study, and so are not publicly available. Data are however available from the authors upon reasonable request and with permission of Division of Geriatric Medicine, Lund University (PI Sölve Elmståhl).

## Abbreviations

CKD-EPI: Chronic kidney disease epidemiology collaboration
CPRS: Comprehensive psychopathological rating scale
CSVD: Cerebral small vessel disease
eGFR: Estimated glomerular filtration rate
GÅS: Good Aging in Skåne
GFR: Glomerular filtration rate
IDMS: Isotope-dilution mass spectrometry
MCI: Mild cognitive impairment
MMSE: Mini mental state examination
SD: Standard deviation
TMT: Trail making test
