# Supplementary material for: Impaired kidney function is associated with lower cognitive function in the elder general population. Results from the Good Aging in Skåne (GÅS) cohort study
Source: BMC Geriatr. 2019 Dec 19;19:360. doi: 10.1186/s12877-019-1381-y (PMC6924030; doi:10.1186/s12877-019-1381-y)
Supplement: Supplementary file 1 — Additional file 1. Characteristics of included individuals versus drop outs in the study. [file 12877_2019_1381_MOESM1_ESM.docx]

| **Additional file 1**. Characteristics of included individuals versus drop outs in the study. | | | | | |
| --- | --- | --- | --- | --- | --- |
| Variable | Groups divided by inclusion in the study | | | | p-value |
|  | Included | Drop outs | | |  |
| Number | 2431 | 500 | | |  |
| eGFR (mL/min/1.73 m2) | 68.1 ± 19.2 | 54.5 ± 19.4 | | | <0.001 |
| Age (years) | 71.4 ± 10.3 | 78.9 ± 10.8 | | | <0.001 |
| Sex |  |  | | | 0.001 |
| women | 1324 (54.5) | 312 (62.4) | | |  |
| men | 1107 (45.5) | 188 (37.6) | | |  |
| Education |  |  | | | <0.001 |
| elementary school not completed | 70 (2.9) | 21 (5.5) | | |  |
| elementary school | 1225 (50.4) | 230 (60.4) | | |  |
| secondary school | 682 (28.1) | 90 (23.6) | | |  |
| >= 1 year extra or university degree | 454 (18.7) | 40 (10.5) | | |  |
| Missing | 0 | 119 | | |  |
| Country of origin |  |  | | | 0.008 |
| Sweden | 2183 (89.8) | 468 (93.6) | | |  |
| other than Sweden | 248 (10.2) | 32 (6.4) | | |  |
| Living |  |  |  | <0.001 | |
| in urban environment | 2179 (89.6) | 323 (79.6) | | |  |
| in rural environment | 252 (10.4) | 83 (20.4) | | |  |
| Missing | 0 | 94 | | |  |
| Cardiovascular risk factors |  |  | | |  |
| Hypertension |  |  | | | <0.001 |
| Yes | 668 (27.6) | 181 (37.5) | | |  |
| No | 1754 (72.4) | 302 (62.5) | | |  |
| Missing | 9 | 17 | | |  |
| Smoking |  |  | | | 0.01 |
| Active smoker | 422 (17.4) | 52 (13.3) | | |  |
| Former smoker | 932 (38.4) | 135 (34.6) | | |  |
| Never smoked | 1073 (44.2) | 203 (52.1) | | |  |
| Missing | 4 | 110 | | |  |
| Diabetes type 1 |  |  | | | 0.013 |
| Yes | 20 (0.8) | 10 (2.1) | | |  |
| No | 2402 (99.2) | 471 (97.9) | | |  |
| Missing | 9 | 19 | | |  |
| Diabetes type 2 |  |  | | | 0.045 |
| Yes | 158 (6.5) | 44 (9.0) | | |  |
| No | 2271 (93.5) | 443 (91.0) | | |  |
| Missing | 2 | 13 | | |  |
| Values above represent number of participants. Values within parenteses represent percentage.  Pearson Chi-Square test was used for all variables except for age and eGFR, where Mann-Whitney test was used.  Values following ± represent SD. Abbreviations: eGFR = estimated glomerular filtration rate, SD = standard deviation. | | | | | |
