# Supplementary material for: Impaired kidney function is associated with lower cognitive function in the elder general population. Results from the Good Aging in Skåne (GÅS) cohort study
Source: BMC Geriatr. 2019 Dec 19;19:360. doi: 10.1186/s12877-019-1381-y (PMC6924030; doi:10.1186/s12877-019-1381-y)
Supplement: Supplementary file 2 — Additional file 2. Results of the cognitive tests in relation to two groups based on eGFR and age. [file 12877_2019_1381_MOESM2_ESM.docx]

| **Additional file 2.** Results of the cognitive tests in relation to two groups based on eGFR and age. | | | | | | | | |
| --- | --- | --- | --- | --- | --- | --- | --- | --- |
| Cognitive test | Age group  (years) | Number of  participants | | | Mean result of  cognitive test | B-coefficient | 95 % CI for B | P-value |
| MMSE | ≤69 | 1255 | | | 27.64 | 0.519 | 0.097, 0.942 | 0.016 |
|  | 70-79 | 464 | | | 26.65 | 0.475 | 0.015, 0.934 | 0.043 |
|  | 80-89 | 552 | | | 25.68 | 0.284 | -0.270, 0.838 | 0.315 |
|  | ≥90 | 131 | | | 25.06 | 0.925 | -1.004, 2.854 | 0.344 |
| Digit span forward | ≤69 | 1251 | | | 6.54 | 0.227 | -0.146, 0.601 | 0.233 |
|  | 70-79 | 462 | | | 6.34 | -0.265 | -0.590, 0.060 | 0.109 |
|  | 80-89 | 544 | | | 5.84 | 0.055 | -0.266, 0.376 | 0.735 |
|  | ≥90 | 122 | | | 5.39 | 0.982 | 0.089, 1.876 | 0.032 |
| Free recall | ≤69 | 1234 | | | 7.38 | 0.274 | -0.189, 0.737 | 0.246 |
|  | 70-79 | 458 | | | 6.38 | 0.457 | 0.057, 0.856 | 0.025 |
|  | 80-89 | 525 | | | 5.52 | 0.051 | -0.340, 0.442 | 0.799 |
|  | ≥90 | 112 | | | 4.94 | -0.375 | -1.831, 1.080 | 0.610 |
| Recognition | ≤69 | 1229 | | | 11.94 | 1.108 | 0.531, 1.686 | <0.001 |
|  | 70-79 | 455 | | | 11.64 | 0.075 | -0.477, 0.627 | 0.791 |
|  | 80-89 | 505 | | | 10.92 | 0.443 | -0.186, 1.073 | 0.167 |
|  | ≥90 | 110 | | | 9.97 | 1.092 | -1.506, 3.689 | 0.407 |
| Word fluency | ≤69 | 1241 | | | 12.86 | 0.833 | -0.085, 1.752 | 0.075 |
|  | 70-79 | 464 | | | 11.34 | 0.819 | 0.025, 1.614 | 0.043 |
|  | 80-89 | 546 | | | 10.70 | 0.311 | -0.502, 1.124 | 0.453 |
|  | ≥90 | 125 | | | 10.34 | 1.254 | -1.373, 3.882 | 0.347 |
| Digit cancellation | ≤69 | 1255 | | | 18.47 | 1.105 | 0.317, 1.892 | 0.006 |
|  | 70-79 | 454 | | | 16.29 | 0.514 | -0.203, 1.231 | 0.160 |
|  | 80-89 | 509 | | | 13.93 | 0.597 | -0.086, 1.280 | 0.087 |
|  | ≥90 | 101 | | | 12.16 | -0.314 | -2.769, 2.141 | 0.800 |
| Pattern comparison | ≤69 | 1248 | | | 29.90 | 2.327 | 1.004, 3.650 | 0.001 |
|  | 70-79 | 453 | | | 24.34 | 1.806 | 0.538, 3.074 | 0.005 |
|  | 80-89 | 497 | | | 20.00 | 0.666 | -0.506, 1.839 | 0.265 |
|  | ≥90 | 92 | | | 17.18 | 1.604 | -2.651, 5.859 | 0.456 |
| TMT B-A | ≤69 | 1083 | | | 13.65 | -6.089 | -8.685, -3.493 | <0.001 |
|  | 70-79 | 345 | | | 20.61 | -3.548 | -8.129, 1.003 | 0.129 |
|  | 80-89 | 344 | | | 25.62 | -3.111 | -8.094, 1.872 | 0.220 |
|  | ≥90 | 64 | | | 38.83 | -5.885 | -29.897, 18.128 | 0.626 |
| Digit span backwards | ≤69 | 1248 | | | 5.68 | 0.366 | -0.034, 0.766 | 0.073 |
|  | 70-79 | 460 | | | 5.18 | 0.060 | -0.284, 0.404 | 0.731 |
|  | 80-89 | 544 | | | 4.83 | 0.092 | -0.233, 0.417 | 0.580 |
|  | ≥90 | 121 | | | 4.44 | 0.485 | -0.554, 1.524 | 0.357 |
| Mental rotations | ≤69 | 1232 | | | 0.63 | 0.043 | 0.005, 0.081 | 0.025 |
|  | 70-79 | 445 | | | 0.58 | -0.012 | -0.047, 0.022 | 0.472 |
|  | 80-89 | 489 | | | 0.55 | -0.011 | -0.042, 0.020 | 0.480 |
|  | ≥90 | 96 | | | 0.50 | 0.057 | -0.037, 0.151 | 0.233 |
| Confidence judgement | ≤69 | 1237 | | | 0.11 | -0.002 | -0.019, 0.015 | 0.825 |
|  | 70-79 | 459 | | | 0.10 | 0.003 | -0.013, 0.018 | 0.741 |
|  | 80-89 | 529 | | | 0.10 | <0.001 | -0.015, 0.014 | 0.965 |
|  | ≥90 | 118 | | | 0.12 | -0.051 | -0.106, 0.004 | 0.071 |
| Multiple linear regression models of cognitive tests in relation to kidney function divided into two groups, impaired kidney function (eGFR <60 mL/min/1.73 m²) and normal kidney function (eGFR ≥60 mL/min/1.73 m²). Age divided into four groups, ≤69 years, 70-79 years, 80-89 years, and ≥90 years. All analyses were adjusted for age, sex, education and country of origin. Abbreviations: eGFR = estimated glomerular filtration rate, CI = confidence interval. | | | | | | | | |
|  | | |  |  |  |  |  |  |
