# Supplementary material for: Impaired kidney function is associated with lower cognitive function in the elder general population. Results from the Good Aging in Skåne (GÅS) cohort study
Source: BMC Geriatr. 2019 Dec 19;19:360. doi: 10.1186/s12877-019-1381-y (PMC6924030; doi:10.1186/s12877-019-1381-y)
Supplement: Supplementary file 3 — Additional file 3. Results of the cognitive tests in relation to four groups based on eGFR and age. [file 12877_2019_1381_MOESM3_ESM.docx]

| **Additional file 3.** Results of the cognitive tests in relation to four groups based on eGFR and age. | | | | | | | |
| --- | --- | --- | --- | --- | --- | --- | --- |
| Cognitive test | Age group (years) | Number of participants | Mean result of cognitive test | eGFR level compared to eGFR ≥60 mL/min/1.73 m² as a reference | B-coefficient | 95 % CI for B | P-value |
| MMSE | ≤69 | 1255 | 27.64 | <30 30-<45 45-<60 | -0,873 -0,592 -0,482 | -2.527, 0.782 -1.765, 0.580 -0.947, -0.018 | 0.301 0.322 0.042 |
|  | 70-79 | 464 | 26.65 | <30 30-<45 45-<60 | -0,857 -0,892 -0,327 | -2.315, 0.602 -1.762, -0.022 -0.837, .0182 | 0.249 0.045 0.207 |
|  | 80-89 | 552 | 25.68 | <30 30-<45 45-<60 | -0,999 -0,587 0,012 | -2.120, 0.122 -1.268, 0.094 -0.598, 0.622 | 0.081 0.091 0.970 |
|  | ≥90 | 131 | 25.06 | <30 30-<45 45-<60 | -0,643 -1,175 -0,815 | -3.031, 1.744 -3.263, 0.913 -2.879, 1.248 | 0.595 0.268 0.436 |
| Digit span forward | <=69 | 1251 | 6.54 | <30 30-<45 45-<60 | 0,535 -0,738 -0,214 | -0.914, 1.984 -1.810, 0.334 -0.623, 0.195 | 0.469 0.177 0.304 |
|  | 70-79 | 462 | 6.34 | <30 30-<45 45-<60 | 0,036 -0,111 0,388 | -0.991, 1.064 -0.724, 0.502 0.028, 0.748 | 0.945 0.722 0.035 |
|  | 80-89 | 544 | 5.84 | <30 30-<45 45-<60 | -0,158 -0,061 -0,037 | -0.815, 0.498 -0.456, 0.335 -0.391, 0.318 | 0.636 0.762 0.839 |
|  | >=90 | 122 | 5.39 | <30 30-<45 45-<60 | -0,982 -0,718 -1,231 | -2.056, 0.093 -1.673, 0.238 -2.182, -0.280 | 0.073 0.139 0.012 |
| Free recall | ≤69 | 1234 | 7.38 | <30 30-<45 45-<60 | 0,861 -1,106 -0,233 | -0.924, 2.646 -2.371, 0.159 -0.744, 0.277 | 0.344 0.087 0.370 |
|  | 70-79 | 458 | 6.38 | <30 30-<45 45-<60 | -0,563 -1,089 -0,279 | -1.876, 0.750 -1.839, -0.339 -0.719, 0.161 | 0.400 0.005 0.214 |
|  | 80-89 | 525 | 5.52 | <30 30-<45 45-<60 | -0,136 -0,042 -0,045 | -0.963, 0.690 -0.528, 0.444 -0.477, 0.387 | 0.746 0.866 0.839 |
|  | >=90 | 112 | 4.94 | <30 30-<45 45-<60 | -0,193 0,013 0,933 | -1.984, 1.598 -1.540, 1.565 -0.606, 2.473 | 0.831 0.987 0.232 |
| Recognition | ≤69 | 1229 | 11.94 | <30 30-<45 45-<60 | -0,183 -1,809 -1,071 | -2.397, 2.031 -3.378, -0.239 -1.708, -0.434 | 0.871 0.024 0.001 |
|  | 70-79 | 455 | 11.64 | <30 30-<45 45-<60 | -0,795 -0,302 0,050 | -2.605, 1.016 -1.336, 0.733 -0.563, 0.663 | 0.389 0.567 0.872 |
|  | 80-89 | 505 | 10.92 | <30 30-<45 45-<60 | -0,352 -0,362 -0,507 | -1.685, 0.981 -1.143, 0.418 -1.205, 0.191 | 0.604 0.362 0.154 |
|  | ≥90 | 110 | 9.97 | <30 30-<45 45-<60 | -2,271 -1,775 0,090 | -5.396, 0.855 -4.537, 0.988 -2.655, 2.834 | 0.153 0.206 0.948 |
| Word fluency | ≤69 | 1241 | 12.86 | <30  30-<45  45-<60 | 1,285-2,762-0,700 | -2.587, 5.157  -5.378, -0.145-1.699, 0.300 | 0.515  0.039  0.170 |
|  | 70-79 | 464 | 11.34 | <30 30-<45 45-<60 | -2,248 -0,511 -0,772 | -4.766, 0.271 -1.994, 0.972 -1.656, 0.111 | 0.080 0.499 0.087 |
|  | 80-89 | 546 | 10.70 | <30 30-<45 45-<60 | -0,060 -0,511 -0,220 | -1.778, 1.657 -1.515, 0.492 -1.118, 0.678 | 0.945 0.317 0.631 |
|  | ≥90 | 125 | 10.34 | <30 30-<45 45-<60 | -1,108 -1,266 -1,306 | -4.310, 2.095 -4.100, 1.569 -4.130, 1.518 | 0.495 0.378 0.362 |
| Digit cancellation | ≤69 | 1255 | 18.47 | <30 30-<45 45-<60 | 0,068 -2,851 -0,918 | -3.286, 3.421 -5.022, -0.679 -1.778, -0.058 | 0.968 0.010 0.036 |
|  | 70-79 | 454 | 16.29 | <30 30-<45 45-<60 | -1,027 -1,338 -0,262 | -3.273, 1.219 -2.738, 0.063 -1.054, 0.529 | 0.369 0.061 0.515 |
|  | 80-89 | 509 | 13.93 | <30 30-<45 45-<60 | -1,042 -0,543 -0,569 | -2.487, 0.403 -1.393, 0.306 -1.322, 0.183 | 0.157 0.210 0.138 |
|  | ≥90 | 101 | 12.16 | <30 30-<45 45-<60 | 1,440 -0,363 0,377 | -1.580, 4.459 -3.034, 2.308 -2.246, 3.000 | 0.346 0.788 0.776 |
| Pattern comparison | ≤69 | 1248 | 29.90 | <30 30-<45 45-<60 | 3,860 -4,437 -2,397 | -1.740, 9.461 -8.064, -0.811 -3.842, -0.952 | 0.177 0.017 0.001 |
|  | 70-79 | 453 | 24.34 | <30 30-<45 45-<60 | -2,384 -4,005 -1,249 | -6.337, 1.569 -6.549, -1.461 -2.639, 0.140 | 0.236 0.002 0.078 |
|  | 80-89 | 497 | 20.00 | <30 30-<45 45-<60 | -0,980 -1,734 0,026 | -3.459, 1.498 -3.180, -0.287 -1.260, 1.312 | 0.437 0.019 0.969 |
|  | ≥90 | 92 | 17.18 | <30 30-<45 45-<60 | -1,531 -2,751 -0,550 | -6.773, 3.711 -7.367, 1.864 -5.150, 4.050 | 0.563 0.239 0.813 |
| TMT B-A | ≤69 | 1083 | 13.65 | <30 30-<45 45-<60 | -4,452 3,060 7,332 | -14.571, 5.667 -4.112, 10.231 4.483, 10.181 | 0.388 0.403 <0.001 |
|  | 70-79 | 345 | 20.61 | <30 30-<45 45-<60 | -1,614 8,240 2,391 | -18.529, 15.300 0.159, 16.320 -2.737, 7.520 | 0.851 0.046 0.360 |
|  | 80-89 | 344 | 25.62 | <30 30-<45 45-<60 | -1,777 3,417 3,520 | -12.934, 9.380 -3.115, 9.948 -1.904, 8.944 | 0.754 0.304 0.203 |
|  | ≥90 | 64 | 38.83 | <30 30-<45 45-<60 | 15,338 5,692 1,426 | -15.352, 46.028 -20.380, 31.764 -25.061, 27.913 | 0.321 0.664 0.915 |
| Digit span backwards | ≤69 | 1248 | 5.68 | <30 30-<45 45-<60 | -0,107 -0,975 -0,309 | -1.651, 1.436 -2.171, 0.222 -.0745, 0.127 | 0.892 0.110 0.165 |
|  | 70-79 | 460 | 5.18 | <30 30-<45 45-<60 | 0,404 -0,482 0,003 | -0.679, 1.487 -1.146, 0.183 -0.376, 0.383 | 0.464 0.155 0.986 |
|  | 80-89 | 544 | 4.83 | <30 30-<45 45-<60 | -0,245 -0,333 0,082 | -0.906, 0.416 -0.731, 0.065 -0.276, 0.439 | 0.466 0.101 0.654 |
|  | ≥90 | 121 | 4.44 | <30 30-<45 45-<60 | -1,008 0,065 -0,773 | -2.230, 0.215 -1.022, 1.151 -1.856, 0.310 | 0.105 0.906 0.160 |
| Mental rotations | ≤69 | 1232 | 0.63 | <30 30-<45 45-<60 | -0,006 -0,037 -0,047 | -0.167, 0.154 -0.141, 0.067 -0.088, -0.005 | 0.938 0.484 0.028 |
|  | 70-79 | 445 | 0.58 | <30 30-<45 45-<60 | 0,037 0,012 0,011 | -0.074, 0.148 -0.058, 0.081 -0.027, 0.048 | 0.510 0.744 0.578 |
|  | 80-89 | 489 | 0.55 | <30 30-<45 45-<60 | 0,017 <0,001 0,017 | -0.051, 0.085 -0.038, 0.039 -0.017, 0.052 | 0.618 0.990 0.325 |
|  | ≥90 | 96 | 0.50 | <30 30-<45 45-<60 | -0,025 -0,055 -0,073 | -0.142, 0.091 -0.160, 0.049 -0.176, 0.029 | 0.665 0.296 0.159 |
| Confidence judgement | ≤69 | 1237 | 0.11 | <30 30-<45 45-<60 | -0,041 -0,023 0,009 | -0.108, 0.025 -0.070, 0.024 -0.010, 0.027 | 0.226 0.340 0.357 |
|  | 70-79 | 459 | 0.10 | <30 30-<45 45-<60 | -0,004 0,022 -0,009 | -0.053, 0.046 -0.007, 0.052 -0.027, 0.008 | 0.875 0.136 0.295 |
|  | 80-89 | 529 | 0.10 | <30 30-<45 45-<60 | -0,005 -0,003 0,003 | -0.035, 0.025 -0.021, 0.015 -0.013, 0.019 | 0.736 0.770 0.712 |
|  | ≥90 | 118 | 0.12 | <30 30-<45 45-<60 | 0,064 0,043 0,053 | -0.004, 0.131 -0.016, 0.103 -0.006, 0.111 | 0.064 0.151 0.078 |
| Multiple linear regression models of cognitive tests in relation to kidney function divided into four groups, severely impaired (eGFR <30 mL/min/1.73 m²), moderately impaired (eGFR 30-<45 mL/min/1.73 m²), mildly impaired (eGFR 45-<60 mL/min/1.73 m²), and normal kidney function (eGFR ≥60 mL/min/1.73 m²), with eGFR ≥60 mL/min/1.73 m² as reference. Age divided into four groups, ≤69 years, 70-79 years, 80-89 years, and ≥90 years. All analyses were adjusted for age, sex, education and country of origin. Abbreviations: eGFR = estimated glomerular filtration rate, CI = confidence interval. | | | | | | | |
