# Supplementary material for: Impaired kidney function is associated with lower cognitive function in the elder general population. Results from the Good Aging in Skåne (GÅS) cohort study
Source: BMC Geriatr. 2019 Dec 19;19:360. doi: 10.1186/s12877-019-1381-y (PMC6924030; doi:10.1186/s12877-019-1381-y)
Supplement: Supplementary file 4 — Additional file 4. Interaction analysis model. [file 12877_2019_1381_MOESM4_ESM.docx]

| **Additional file 4.** Interaction analysis model. | |  |  |
| --- | --- | --- | --- |
| Cognitive test | Interaction variable | p-value for interaction  variable | Interaction |
| MMSE | age sex education country of origin | 0.165 0.767 0.370 0.005 | No No No Yes |
| Digit span forward | age sex education country of origin | 0.146 0.412 0.949 0.065 | No No No No |
| Free recall | age sex education country of origin | 0.787 0.500 0.301 0.073 | No No No No |
| Recognition | age sex education country of origin | 0.432 0.303 0.802 0.282 | No No No No |
| Word fluency | age sex education country of origin | 0.616 0.793 0.270 0.028 | No No No Yes |
| Digit cancellation | age sex education country of origin | 0.329 0.245 0.351 0.615 | No No No No |
| Pattern comparison | age sex education country of origin | 0.405 0.781 0.981 0.125 | No No No No |
| TMT B-A | age sex education country of origin | 0.326 0.603 0.101 0.327 | No No No No |
| Digit span backwards | age sex education country of origin | 0.765 0.027 0.495 0.010 | No Yes No Yes |
| Mental rotations | age sex education country of origin | 0.144 0.021 0.007 0.121 | No Yes Yes No |
| Confidence judgement | age sex education country of origin | 0.044 0.216 0.981 0.113 | Yes No No No |
| Multiple linear regression models of cognitive tests in relation to eGFR (<60 mL/min/1.73 m² and ≥60 mL/min/1.73 m²), age, sex, education and country of origin. Interaction analyses between eGFR (<60 mL/min/1.73 m² and ≥60 mL/min/1.73 m²) and the demographic variables age, sex, education and country of origin. Abbreviations: eGFR = estimated glomerular filtration rate. | | | |
