# Supplementary material for: Impaired kidney function is associated with lower cognitive function in the elder general population. Results from the Good Aging in Skåne (GÅS) cohort study
Source: BMC Geriatr. 2019 Dec 19;19:360. doi: 10.1186/s12877-019-1381-y (PMC6924030; doi:10.1186/s12877-019-1381-y)
Supplement: Supplementary file 5 — Additional file 5. Results of the cognitive tests in relation to two groups based on eGFR, including interaction. [file 12877_2019_1381_MOESM5_ESM.docx]

| **Additional file 5.** Results of the cognitive tests in relation to two groups based on eGFR, including interaction. | | | | | | | | |
| --- | --- | --- | --- | --- | --- | --- | --- | --- |
| Cognitive test | Cognitive domain | Number of participants | Mean test result | Variable interacting with eGFR | | B-coefficient | 95% CI for B | p-value |
|  |  |  |  |  |  |  |  |  |
| MMSE | Global | 2402 | 26.86 | country of origin | born in Sweden born in other country | 0.505 -0.621 | 0.226, 0.783 -1.391, 0.150 | <0.001 0.115 |
| Word fluency | Language | 2376 | 11.94 | country of origin | born in Sweden born in other country | 0.799 -0.810 | 0.300, 1.298 -2.195, 0.575 | 0.002 0.252 |
| Digit span backwards | Executive function | 2373 | 5.33 | sex | man woman | 0.354 -0.004 | 0.079, 0.630 -0.253, 0.246 | 0.012 0.977 |
| Digit span backwards | Executive function | 2373 | 5.33 | country of origin | born in Sweden born in other country | 0.219 -0.561 | 0.006, 0.431 -1.146, 0.029 | 0.044 0.060 |
| Mental rotations | Perceptual-motor | 2262 | 0.6 | sex | man woman | 0.022 -0.015 | -0.004, 0.049 -0.040, 0.009 | 0.100 0.219 |
| Mental rotations | Perceptual-motor | 2262 | 0.6 | education | elementary school not completed fulfilled elementary school fulfilled secondary school higher education | -0.079 -0.018 0.049 0.004 | -0.167, 0.009 -0.042, 0.007 0.016, 0,082 -0.039, 0.048 | 0.079 0.159 0.003 0.841 |
| Confidence judgement | Meta-memory | 2343 | 0.066 | age | effect per year (normal eGFR) effect per year (impaired eGFR) | -0.0009 -0.0002 | -0.0013, -0.0005 -0.0007, 0.0007 | <0.001 0.959 |
| Multiple linear regression models of cognitive tests in relation to kidney function divided into two groups, impaired kidney function (eGFR <60 mL/min/1.73 m²) and normal kidney function (eGFR ≥60 mL/min/1.73 m²). All analyses were adjusted for age, sex, education, country of origin and interaction variable(s). Abbreviations: eGFR = estimated glomerular filtration rate, CI = confidence interval. | | | | | | | | |
